# Protocol for the *in vitro* reconstruction of site-specifically phosphorylated RNA Pol II to identify the recruitment of novel transcription regulators

**DOI:** 10.1016/j.xpro.2024.103277

**Published:** 2024-08-27

**Authors:** Haley A. Hardtke, Rosamaria Y. Moreno, Y. Jessie Zhang

**Affiliations:** 1 Department of Molecular Biosciences, University of Texas, Austin, TX 78712, USA

**Keywords:** Protein Biochemistry, Protein expression and purification, Proteomics

## Abstract

The repetitive C-terminal domain (CTD) of the largest subunit of RNA polymerase II (RNAPII) becomes differentially phosphorylated throughout the transcription cycle. Here, we present a protocol to site-specifically phosphorylate the CTD of RNAPII by leveraging the specificity of well-characterized CTD kinases. We describe the steps for optimal phosphorylation of the CTD and the preparation of nuclear protein extract. This protocol can be used to identify the interactome of a phospho-CTD and has the potential to identify novel RNAPII-binding proteins.

For complete details on the use and execution of this protocol, please refer to Moreno et al.^1^

## Highlights

• Purify the unphosphorylated C-terminal domain (CTD) of RNA polymerase II
• Site-specifically phosphorylate residues from CTD heptad repeats
• Identify the interactome of a CTD with specific phosphorylation sites

## Abstract (editor highlights)

Publisher’s note: Undertaking any experimental protocol requires adherence to local institutional guidelines for laboratory safety and ethics.

## Graphical abstract

**Figure:**
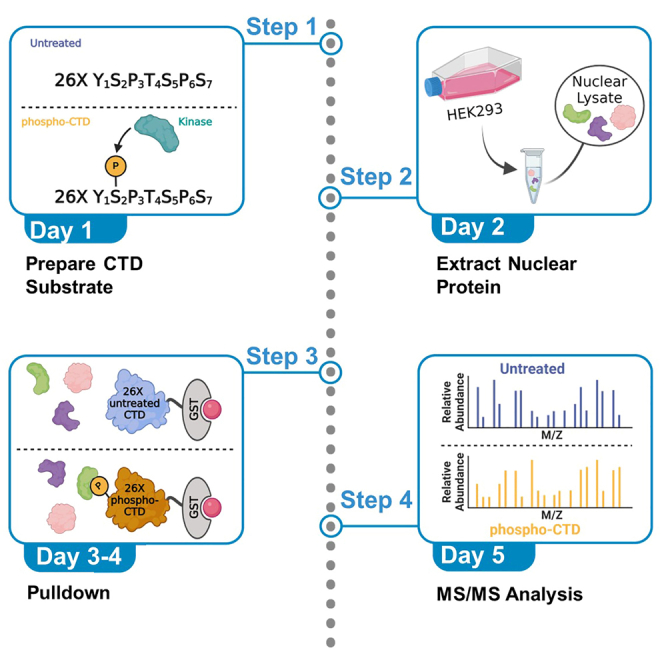

## Before you begin

This protocol seeks to identify the interactomes for the different phosphorylation states of the C-terminal domain (CTD) of RNA polymerase II (RNAPII). The CTD of RNAPII is composed of tandem heptad repeats of the consensus sequence: YSPTSPS.^2^ Five of the seven consensus residues are post-translationally modified by phosphorylation throughout the transcription cycle. Thus, the CTD of RNAPII serves as a critical binding platform for the recruitment of proteins that can regulate progression of transcription.^3^

Many groups have sought to characterize RNAPII protein-protein interactions, and as a result a myriad of different approaches have emerged to identify RNAPII interactomes at different stages of transcription.^4^ Affinity purification mass spectrometry (AP-MS) is one such method that has been leveraged to identify RNAPII-binding proteins. Traditionally, this strategy uses an antibody against a suspected RNAPII-binding protein to do the pull-down experiment. If the suspected RNAPII-binding protein does associate with the transcription complex, subunits of RNAPII should be top hits in the pull-down results. Though successful in identifying some RNAPII-binding proteins, such as Set2 (yeast) and Rtr1 (yeast), this approach doesn’t distinguish interactions with different phosphorylated RNAPII species.^5^^,^^6^^,^^7^^,^^8^^,^^9^^,^^10^ Another strategy employed to characterize the RNAPII interactome was to immunopreciptate the RNAPII complex from cell lysate and then subsequently immunopreciptate out different RNAPII complexes using antibodies that are specific to different CTD phosphorylation signatures.^11^^,^^12^ This method has been used to characterize the interactomes of RNAPII complexes phosphorylated at either the Y1, S2, T4, S5, or S7 positions in yeast. However, the scope of this strategy is dominated by the recognition profile of phospho-specific CTD antibodies, which often struggle to recognize the CTD in a site-specific manner when neighboring residues are post translationally modified.^13^^,^^14^ Furthermore, since RNAPII is hyperphosphorylated during transcription, it will be difficult to discern which proteins are recruited by specific phosphorylation signatures. Another strategy that has been used with some success has been to mutate the CTD in some manner, disrupting site-specific phosphorylation, and use the mutant CTD in a pull-down experiment to better understand the role of different phosphorylation sites in recruiting proteins to the RNAPII complex during transcription.^15^ One final strategy that has been used to characterize the RNAPII interactome is to use either synthetic phospho-CTD peptides or an *in vitro* phosphorylated CTD construct as bait in a pull-down experiment.^16^^,^^17^^,^^18^ Our strategy fits into this final category. In this protocol a GST-tagged yeast CTD, composed entirely of consensus repeats, is phosphorylated with kinases of known specificity. The phosphorylated CTD is subsequently used in a label-free proteomics experiment where the "bait" phospho-CTD is incubated with HEK293 nuclear lysate. The advantage of the strategy employed in this protocol is that we can directly link protein recruitment to a specific RNAPII post translational modification, since we are using a homogenously phosphorylated substrate. Furthermore, the hyperphosphorylated long CTD mimics the physiological state of the CTD during transcription and greatly increases the signal/noise ratio for a more confident identification of phospho-RNAPII interactomes.

The *in vitro* specificity of the kinases towards the CTD that are used in this study (ERK2, TFIIH and DYRK1a) have been characterized using mass spectrometry.^19^^,^^20^^,^^21^^,^^22^ Though we used HEK293 cells for our experiments and reconstructed Ser2 and Ser5 phosphorylation on the CTD *in vitro*, this protocol can be expanded to other phosphorylation sites, cell lines, or species. Additionally, this protocol can be adapted if other CTD kinases are characterized and can be used to identify the interactomes for different CTD constructs (i.e., distal vs. proximal CTD, or CTDs of varying lengths, etc.).

### Cell culture

**Timing: 7 days**

1. Culture cells for the pull-down experiment. Ensure that the cells have been in culture for a few passages and are fully equilibrated with media and culture conditions prior to starting the pull-down experiment.
  a. For HEK293 cells approximately 200 million cells are needed for one pull-down experiment, this is the equivalent of approximately 6 confluent T175 flasks. At minimum, 50 million cells per sample are needed. Thus, you can have a maximum of 4 samples per pull-down experiment.
  b. Seed 1 extra flask to count and estimate cell viability.
2. Detach cells from the culture dish.
  a. Discard medium from the flask (or culture dish).
  b. Wash cells with sterile 1× PBS equilibrated to 20°C–22°C.
  c. Add Trypsin-EDTA to flask and place flask in incubator at 37°C for a maximum of 2 min.
  d. Use microscope to ensure cells have fully detached from the flask.
  e. Quench trypsin-EDTA with DMEM complete media.
    ***Note:*** Ensure the final volume of trypsin/DMEM is 50 mL or less so that the entire solution fits into a 50 mL conical vial that can be placed into a centrifuge to pellet cells.
  f. Pellet cells by centrifuging for 5 min at 200 g at 20°C–22°C.
3. Wash cells.
  a. Carefully resuspend cells in 1 mL of ice-cold sterile 1× PBS.
  b. Transfer cell suspension to a 2 mL tube.
  c. Pellet cells by centrifuging for 5 min at 200 g at 4°C.
  d. Determine packed cell volume (PCV). This is the volume that the pelleted cells take up in the tube. The PCV will be used as the volume for wash steps during protein extraction steps.
    ***Note:*** In our experience the PCV is typically about 1 mL.
  e. Discard supernatant, and carefully resuspend cells in 1 mL ice-cold sterile 1× PBS for a second wash step.
  f. If using the cells directly pellet cells as described in step 3c and proceed to nuclear protein extraction steps detailed in steps 18–20 in the step-by-step method details section.
    ***Note:*** Freeze cells at −20°C if you are not going to extract protein immediately. It is recommended to use cells immediately after harvesting.

## Key resources table

**Table:** 

REAGENT or RESOURCE	SOURCE	IDENTIFIER
**Chemicals, peptides, and recombinant proteins**		

TFIIH (Cdk7/CyclinH/MAT1 (CAK complex))	Millipore	Cat #14-476
Tris base	Fisher Scientific	Cat #BP152-5; CAS 77-86-1
NaCl	Fisher Scientific	Cat #BP358-212; CAS 7647-14-5
Imidazole	Acros Organics	Cat #122020020; CAS 288-32-4
Glycerol	Fisher Scientific	Cat #G33-4; CAS 56-81-5
2-Mercaptoethanol	Acros Organics	Cat #125472500; CAS 60-24-2
Triton X-100	United States Biomedical	Cat #22686; 9036-19-4
MgCl_2_	MP Biomedicals	Cat #194698; CAS 7791-18-6
HEPES	MilliporeSigma	Cat #H-3375; CAS 7365-45-9
Sucrose	Fisher Scientific	Cat#AA36508A1; CAS 57-50-1
L-glutathione reduced	Alfa Aesar	Cat# A18014; CAS 70-18-8
Halt protease and phosphatase inhibitor	Thermo Fisher Scientific	Cat #78442
Luria-Bertani broth	MP Biomedicals	Cat #1005361
Terrific broth	Fisher Scientific	Cat #AAJ75856-A1
Yeast extract	Fisher Scientific	Cat #BP9727-500; CAS 8013-01-2
Tryptone	Fisher Scientific	Cat # BP1421-500; CAS 91079-40-2
KCl	ChemPure Chemicals	Cat #831-663; CAS 7447-40-7
IPTG	Gold Biotechnology	Cat #I2481C50; CAS 367-93-1
Ampicillin	MilliporeSigma	Cat #A9518-100G; CAS 69-52-3
Kanamycin	Gold Biotechnology	Cat #K-120-25; CAS 25389-94-0
ATP	Boehringer Mannheim	Lot #1153418
Benzonase	MilliporeSigma	Cat #70746-4; CAS 9025-65-4

**Experimental models: Cell lines**		

HEK293	ATCC	RRID:CVCL_0063

**Recombinant DNA**		

RPB1 cDNA	Addgene	Cat #75284
DYRK1A	Addgene	Cat #79690
ERK2	Addgene	Cat #39212

**Software and algorithms**		

GraphPad Prism (version 10.2.3)	GraphPad Software, LLC	https://www.graphpad.com

**Other**		

Snakeskin dialysis tubing (10,000 MWCO)	Thermo Fisher Scientific	Cat #68100
Ni-NTA agarose	QIAGEN	Cat #30210
GN-6 Metricel membrane disc filters (0.45 μM)	Fisher Scientific	Cat #50-206-3269
Glutathione beads	G-Biosciences	Cat #786-280
Sonicator	Misonix	Model #S-4000
Vivaspin 20, 10 kDa MWCO	MilliporeSigma	Cat #GE28-9323-60
Thermomixer C	Eppendorf	EP5382000023
Roto-Mini Plus	Ward’s Science	Cat #470313-912

## Materials and equipment

### SOC media

Dissolve the following reagents in 40 mL of ddH_2_O:

• 1.00 g tryptone
• 0.25 g yeast extract
• 0.025 g NaCl

Add 500 μL of 250 mM KCl to the solution and dilute the solution to 49 mL. Adjust the pH of the solution to 7.0 with 1 M NaOH. Autoclave the solution, and then let the solution cool to the touch. Add 250 μL of sterile 2 M MgCl_2_ and 1 mL of sterile 1 M glucose. Aliquot 800 μL prepared SOC media into microcentrifuge tubes and freeze at −20°C for future use. SOC media can be stored at −20°C indefinitely.

**Table:** CTD lysis buffer

Reagent	Final concentration	Amount
Tris-HCl pH 7.5 (1,000 mM)	50 mM	50 mL
NaCl (5,000 mM)	500 mM	100 mL
Imidazole (1,000 mM)	20 mM	20 mL
Glycerol (100%)	5%	50 mL
2-Mercaptoethanol (14,300 mM)	10 mM	0.7 mL^∗^
ddH_2_O	N/A	779.3 mL
**Total**	**N/A**	**1,000 mL**

^∗^Add 2-mercaptoethanol directly before use.

CTD lysis buffer can be stored at 20°C–22°C indefinitely. Protect the buffer from light by wrapping it in tin foil to prevent degradation of imidazole. Aliquot the amount of CTD lysis buffer that is needed for individual experiments and supplement the aliquot with the necessary amount of 2-mercaptoethanol. Place the aliquot at 4°C to equilibrate the buffer to purification conditions.

**CRITICAL:** 2-Mercaptoethanol is a skin irritant and is toxic if swallowed or inhaled. Please handle with appropriate lab safety gear in a fume hood.

**Table:** CTD elution buffer

Reagent	Final concentration	Amount
Tris-HCl pH 7.5 (1,000 mM)	50 mM	5 mL
NaCl (5,000 mM)	500 mM	10 mL
Imidazole (1,000 mM)	250 mM	25 mL
Glycerol (100%)	5%	5 mL
2-Mercaptoethanol (14,300 mM)	10 mM	70 μL^∗^
ddH_2_O	N/A	54.93 mL
**Total**	**N/A**	**100 mL**

^∗^Add 2-mercaptoethanol directly before use.

CTD elution buffer can be stored at 20°C–22°C indefinitely. Protect the buffer from light by wrapping it in tin foil to prevent degradation of imidazole. Aliquot the amount of CTD elution buffer that is needed for individual experiments and supplement the aliquot with the necessary amount of 2-mercaptoethanol. Place the aliquot at 4°C to equilibrate the buffer to purification conditions.

**Table:** CTD SEC buffer

Reagent	Final concentration	Amount
Tris-HCl pH 7.5 (1000 mM)	50 mM	50 mL
NaCl (5,000 mM)	500 mM	100 mL
Glycerol (100%)	5%	50 mL
2-Mercaptoethanol (14,300 mM)	10 mM	0.7 mL^∗^
ddH_2_O	N/A	799.3 mL
**Total**	**N/A**	**1,000 mL**

^∗^Add 2-mercaptoethanol directly before use.

Keep CTD SEC buffer at 4°C. Prepare fresh every time and filter the buffer using a 0.45 μM filter after preparing.

**Table:** DYRK1a lysis buffer

Reagent	Final concentration	Amount
Tris-HCl pH 8.0 (1,000 mM)	50 mM	50 mL
NaCl (5,000 mM)	500 mM	100 mL
Imidazole (1,000 mM)	15 mM	15 mL
Glycerol (100%)	10%	100 mL
Triton X-100 (100%)	0.1%	1 mL
2-Mercaptoethanol (14,300 mM)	10 mM	0.7 mL^∗^
ddH_2_O	N/A	733.3 mL
**Total**	**N/A**	**1,000 mL**

^∗^Add 2-mercaptoethanol directly before use.

DYRK1a lysis buffer can be stored at 20°C–22°C indefinitely. Protect the buffer from light by wrapping it in tin foil to prevent degradation of imidazole. Aliquot the amount of DYRK1a lysis buffer that is needed for individual experiments and supplement the aliquot with the necessary amount of 2-mercaptoethanol. Place the aliquot at 4°C to equilibrate the buffer to purification conditions.

**CRITICAL:** Triton X-100 is a skin and eye irritant and is toxic if swallowed. Please handle with appropriate lab safety gear.

**Table:** DYRK1a wash buffer

Reagent	Final concentration	Amount
Tris-HCl pH 8.0 (1,000 mM)	50 mM	25 mL
NaCl (5,000 mM)	500 mM	50 mL
Imidazole (1,000 mM)	25 mM	12.5 mL
2-Mercaptoethanol (14,300 mM)	10 mM	0.35 mL^∗^
ddH_2_O	N/A	412.15 mL
**Total**	**N/A**	**500 mL**

^∗^Add 2-mercaptoethanol directly before use.

DYRK1a wash buffer can be stored at 20°C–22°C indefinitely. Protect the buffer from light by wrapping it in tin foil to prevent degradation of imidazole. Aliquot the amount of DYRK1a wash buffer that is needed for individual experiments and supplement the aliquot with the necessary amount of 2-mercaptoethanol. Place the aliquot at 4°C to equilibrate the buffer to purification conditions.

**Table:** DYRK1a elution buffer

Reagent	Final concentration	Amount
Tris-HCl pH 8.0 (1,000 mM)	50 mM	10 mL
NaCl (5,000 mM)	500 mM	20 mL
Imidazole (1,000 mM)	200 mM	40 mL
2-Mercaptoethanol (14,300 mM)	10 mM	0.14 mL^∗^
ddH_2_O	N/A	129.86 mL
**Total**	**N/A**	**200 mL**

^∗^Add 2-mercaptoethanol directly before use.

DYRK1a elution buffer can be stored at 20°C–22°C indefinitely. Protect the buffer from light by wrapping it in tin foil to prevent degradation of imidazole. Aliquot the amount of DYRK1a elution buffer that is needed for individual experiments and supplement the aliquot with the necessary amount of 2-mercaptoethanol. Place the aliquot at 4°C to equilibrate the buffer to purification conditions.

**Table:** DYRK1a SEC buffer

Reagent	Final concentration	Amount
Tris-HCl pH 8.0 (1,000 mM)	50 mM	50 mL
NaCl (5,000 mM)	200 mM	40 mL
2-Mercaptoethanol (14,300 mM)	10 mM	0.7 mL^∗^
ddH_2_O	N/A	909.3 mL
**Total**	**N/A**	**1,000 mL**

^∗^Add 2-mercaptoethanol directly before use.

Keep DYRK1a SEC buffer at 4°C. Prepare fresh every time and filter the buffer using a 0.45 μM filter after preparing.

**Table:** ERK2 lysis buffer

Reagent	Final concentration	Amount
Tris-HCl pH 8.0 (1,000 mM)	50 mM	50 mL
NaCl (5,000 mM)	500 mM	100 mL
Imidazole (1,000 mM)	15 mM	15 mL
Glycerol (100%)	10%	100 mL
Triton X-100 (100%)	0.1%	1 mL
2-Mercaptoethanol (14,300 mM)	10 mM	0.7 mL^∗^
ddH_2_O	N/A	733.3 mL
**Total**	**N/A**	**1,000 mL**

^∗^Add 2-mercaptoethanol directly before use.

ERK2 lysis buffer can be stored at 20°C–22°C indefinitely. Protect the buffer from light by wrapping it in tin foil to prevent degradation of imidazole. Aliquot the amount of ERK2 lysis buffer that is needed for individual experiments and supplement the aliquot with the necessary amount of 2-mercaptoethanol.

**Table:** ERK2 elution buffer

Reagent	Final concentration	Amount
Tris-HCl pH 8.0 (1,000 mM)	50 mM	10 mL
NaCl (5,000 mM)	500 mM	20 mL
Imidazole (1,000 mM)	200 mM	40 mL
2-Mercaptoethanol (14,300 mM)	10 mM	0.14 mL^∗^
ddH_2_O	N/A	129.86 mL
**Total**	**N/A**	**200 mL**

^∗^Add 2-mercaptoethanol directly before use.

ERK2 elution buffer can be stored at 20°C–22°C indefinitely. Protect the buffer from light by wrapping it in tin foil to prevent degradation of imidazole. Aliquot the amount of ERK2 elution buffer that is needed for individual experiments and supplement the aliquot with the necessary amount of 2-mercaptoethanol. Place the aliquot at 4°C to equilibrate the buffer to purification conditions.

**Table:** ERK2 Mono Q buffer A

Reagent	Final concentration	Amount
Tris-HCl pH 8.0 (1,000 mM)	20 mM	20 mL
NaCl (5,000 mM)	50 mM	10 mL
Glycerol (100%)	10%	100 mL
2-Mercaptoethanol (14,300 mM)	10 mM	0.7 mL^∗^
ddH_2_O	N/A	869.3 mL
**Total**	**N/A**	**1,000 mL**

^∗^Add 2-mercaptoethanol directly before use.

Keep ERK2 Mono Q buffer A at 4°C. Prepare fresh every time and filter the buffer using a 0.45 μM filter after preparing.

**Table:** ERK2 Mono Q buffer B

Reagent	Final concentration	Amount
Tris-HCl pH 8.0 (1,000 mM)	20 mM	20 mL
NaCl (5,000 mM)	1,000 mM	200 mL
Glycerol (100%)	10%	100 mL
2-Mercaptoethanol (14,300 mM)	10 mM	0.7 mL^∗^
ddH_2_O	N/A	679.3 mL
**Total**	**N/A**	**1,000 mL**

^∗^Add 2-mercaptoethanol directly before use.

Keep ERK2 Mono Q buffer B at 4°C. Prepare fresh every time and filter the buffer using a 0.45 μM filter after preparing.

**Table:** 4× kinase reaction buffer

Reagent	Final concentration	Amount
Tris-HCl pH 8.0 (1,000 mM)	200 mM	10 mL
MgCl_2_ (1,000 mM)	40 mM	0.4 mL
ddH_2_O	N/A	39.6 mL
**Total**	**N/A**	**50 mL**

Store at 20°C–22°C indefinitely.

***Alternatives:*** To utilize multiple kinases in substrate treatment, adjust the final concentrations of Tris-HCl and MgCl_2_ in the kinase buffer solution accordingly. For instance, if using two different kinases, prepare a 5× kinase buffer instead of the standard 4× concentration.

### ATP (8 mM)

• Dissolve 20.1 mg of ATP (507.18 g/mol) in 4 mL of ddH_2_O.
• Adjust the solution pH to 7.0 with 1 M Tris-HCl pH 7.5.
• Bring the final volume of the solution to 5 mL.

Aliquot 1 mL of the 8 mM ATP stock into microcentrifuge tubes and store at −20°C for up to 2 years. Thaw ATP as needed. Avoid multiple freeze/thaw cycles.

**Table:** Buffer A

Reagent	Final concentration	Amount
HEPES pH 7.4 (1,000 mM)	10 mM	2 mL
NaCl (5,000 mM)	100 mM	4 mL
Sucrose (342.2 g/mol)	300 mM	20.5 g
MgCl_2_ (1,000 mM)	3 mM	0.6 mL
Triton X-100 (100%)	0.5%	1 mL
Protease and phosphatase inhibitors (100×)	1×	2 mL^∗^
ddH_2_O	N/A	190.4 mL
**Total**	**N/A**	**200 mL**

^∗^Add protease and phosphatase inhibitors (100×) directly before use.

Buffer A can be stored at 20°C–22°C indefinitely. Prior to the experiment, place an aliquot of buffer A on ice and supplement the aliquot with the necessary amount of protease and phosphatase inhibitors.

**Table:** Buffer B

Reagent	Final concentration	Amount
Tris-HCl pH 8.0 (1,000 mM)	10 mM	2 mL
NaCl (5,000 mM)	150 mM	6 mL
Protease and phosphatase inhibitors (100×)	1×	2 mL^∗^
ddH_2_O	N/A	190 mL
**Total**	**N/A**	**200 mL**

^∗^Add protease and phosphatase inhibitors (100×) directly before use.

Buffer B can be stored at 20°C–22°C indefinitely. Prior to the experiment, place an aliquot of buffer B on ice and supplement the aliquot with the necessary amount of protease and phosphatase inhibitors.

**Table:** Buffer C

Reagent	Final concentration	Amount
Tris-HCl pH 8.0 (1,000 mM)	20 mM	4 mL
NaCl (5,000 mM)	150 mM	6 mL
2-Mercaptoethanol (14,300 mM)	10 mM	0.14 mL^∗^
ddH_2_O	N/A	180.26 mL
**Total**	**N/A**	**200 mL**

^∗^Add 2-mercaptoethanol directly before use.

Buffer C can be stored at 20°C–22°C indefinitely. Prior to the experiment, place an aliquot of buffer C on ice and supplement the aliquot with the necessary amount of 2-mercaptoethanol.

**Table:** Low salt wash buffer

Reagent	Final concentration	Amount
Tris-HCl pH 8.0 (1,000 mM)	20 mM	4 mL
NaCl (5,000 mM)	150 mM	6 mL
Glycerol (100%)	10%	20 mL
Triton X-100 (100%)	0.1%	0.2 mL
Protease and phosphatase inhibitors (100×)	1×	2 mL^∗^
ddH_2_O	N/A	167.8 mL
**Total**	**N/A**	**200 mL**

^∗^Add protease and phosphatase inhibitors (100×) directly before use.

Low salt wash buffer can be stored at 20°C–22°C indefinitely. Prior to the experiment, place an aliquot of low salt buffer on ice and supplement the aliquot with the necessary amount of protease and phosphatase inhibitors.

**Table:** High salt wash buffer

Reagent	Final concentration	Amount
Tris-HCl pH 8.0 (1,000 mM)	20 mM	4 mL
NaCl (5,000 mM)	500 mM	20 mL
Glycerol (100%)	10%	20 mL
Triton X-100 (100%)	0.1%	0.2 mL
Protease and phosphatase inhibitors (100×)	1×	2 mL^∗^
ddH_2_O	N/A	153.8 mL
**Total**	**N/A**	**200 mL**

^∗^Add protease and phosphatase inhibitors (100×) directly before use.

High salt wash buffer can be stored at 20°C–22°C indefinitely. Prior to the experiment, place an aliquot of high salt buffer on ice and supplement the aliquot with the necessary amount of protease and phosphatase inhibitors.

### Elution buffer

Supplement 1 mL high salt wash buffer with 20 mM reduced glutathione (stock 1000 mM). No additional protease and phosphatase inhibitors are necessary for this solution.

Prepare the glutathione stock solution fresh every time.

## Step-by-step method details

### Purify HGST-26X CTD

**Timing: 5 days**

This section gives step by step instructions for purifying a 26X CTD recombinant protein tagged with an N-terminal hexahistidine glutathione S-transferase (HGST) that will be used as the substrate for phosphorylation and subsequently used as bait in the pull-down experiment.

1. Transform the HGST-26X CTD pET-28a plasmid into *E. coli* BL21 (DE3) cells.
  a. Add 50 ng of the HGST-26X CTD plasmid to a 50 μL aliquot of *E. coli* BL21 (DE3) cells.
  b. Incubate plasmid-cell solution on ice for 30 min.
  c. Place tube containing the plasmid-cell solution in a water bath set to 42°C for 60 s to heat shock.
  d. Place tube on ice for 5 min after heat shock.
  e. Transfer heat shocked cells to a microcentrifuge tube containing 800 μL SOC media.
  f. Place tube in incubator set to 37°C and 200 rpm and shake sample for 1 h to recover cells.
  g. Spin cells down at 3,000 g for 3 min at 20°C–22°C.
  h. Discard supernatant and resuspend cells in 50 μL of LB media.
  i. Plate cells on LB agar plates that contain 50 μg/mL kanamycin.
  j. Place LB/Kanamycin plates in a 37°C incubator for a minimum of 16 h. Colonies should be visible after 24 h.
2. Express HGST 26X CTD.
  a. Add 50 μL of 50 mg/mL kanamycin to Erlenmeyer flask that contains 50 mL of LB media, so that the final concentration of kanamycin in the LB media is 50 μg/mL.
  b. Inoculate the 50 mL LB/Kanamycin flask with cells from a colony on the plate from step 1j.
  c. Grow starter culture in a shaking incubator for 16–18 h at 37°C with shaking at 200 rpm.
  d. Inoculate two 1 L Luria-Bertani broth (LB) cultures with 10 mL starter culture and 50 μg/mL kanamycin.
  e. Grow 1 L cultures at 37°C with shaking at 200 rpm until the OD reaches 0.6.
  f. Induce cultures by adding IPTG so that the final IPTG concentration in each 1 L culture is 0.5 mM and reduce the temperature of the shaker to 16°C to promote proper protein folding during expression.
  g. Let cultures incubate in the shaker 16–18 h at 16°C and 200 rpm.
  h. Pellet cells by centrifugation at 5,500 g for 25 min at 4°C.

***Note:*** Store pellet at −20°C 16–18 h or flash freeze it for long-term storage at −80°C if immediate purification is not required.

**Figure 1:**
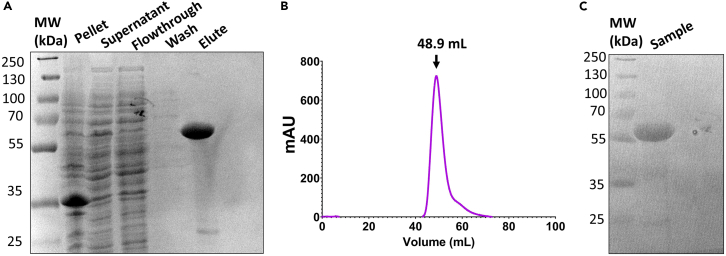
HGST-26X CTD purification (A) SDS-PAGE of HGST-26X CTD purification showing all steps of the nickel-affinity purification. The lanes from left to right are the ladder, pellet (post-sonication), supernatant (post-sonication), nickel column flowthrough, nickel column wash, nickel column elute. Samples were run on a 15% gel. (B) Size-exclusion chromatogram for HGST-26X CTD run on a Superdex 75 column (GE). (C) SDS-PAGE of pooled HGST-26X CTD after running size-exclusion chromatography. Sample was run on a 15% gel.

3. Lyse cells.
  a. Resuspend cells from both pellets in 50 mL CTD lysis buffer.
  b. Sonicate resuspended cells with five 30 s cycles. For each 30 s cycle, set the on time to 1 s, the off time to 5 s, and the amplitude to 90 A. Let the sample rest 3 min on ice between cycles.
    ***Note:*** Maintain the sample on ice throughout the sonication process to prevent overheating.
  c. Pellet cell lysate by centrifugation at 15,000 g for 35 min at 4°C.
4. Purify HGST-26X CTD with nickel affinity resin.
  a. Add 5 mL of Ni-NTA beads to a gravity column.
  b. Equilibrate Ni-NTA beads with 20 column volumes (CV) CTD lysis buffer.
    ***Note:*** Ni-NTA column purification must be done at 4°C.
  c. Run supernatant from step 3c over the equilibrated nickel beads.
  d. Collect the flowthrough and run the flowthrough over the nickel beads a second time.
  e. Wash the column with 10 CV of CTD lysis buffer.
  f. Elute protein with 4 CV of CTD elution buffer.
  g. Run SDS-PAGE to confirm purity. An example SDS-PAGE for all HGST-26X CTD purification steps is shown in Figure 1A. Figure 1C shows HGST-26X CTD purity after size-exclusion chromatography.
5. Further purify HGST-26X CTD by size-exclusion chromatography.
  a. Concentrate eluted protein to 5 mL using a protein concentrator with a molecular weight cut off of 10 kDa.
  b. Buffer exchange eluted protein to CTD SEC buffer by adding 5 mL of SEC buffer to concentrator at a time and reconcentrating the sample to 5 mL.
  c. Repeat step 5b at least 4 additional times to ensure that a minimum of 25 mL of CTD SEC buffer is used to minimize the concentration of imidazole in solution before loading onto the column.
  d. Concentrate the buffer exchanged sample to 2 mL.
  e. Equilibrate Superdex 75 size-exclusion column (GE) with CTD SEC buffer.
  f. Run sample over SEC and pool fractions from peak corresponding to ∼49 kDa as shown in Figure 1B.
  g. Check protein concentration and concentrate to 4 mg/mL.

### Purify DYRK1a kinase

**Timing: 5 days**

This step is an example protocol for purifying an active CTD kinase that will be used to phosphorylate the CTD substrate for the pull-down experiment.

6. Transform the His-DYRK1a (residues 127–485) pET-28a plasmid containing an N-terminal hexahistidine tag into *E. coli* BL21 (DE3) cells.
  a. Add 50 ng of the His-DYRK1a plasmid to a 50 μL aliquot of *E. coli* BL21 (DE3) cells.
  b. Incubate plasmid-cell solution on ice for 30 min.
  c. Place tube containing the plasmid-cell solution in a water bath set to 42°C for 60 s to heat shock.
  d. Place tube on ice for 5 min after heat shock.
  e. Transfer heat shocked cells to a microcentrifuge tube containing 800 μL SOC media.
  f. Place tube in incubator set to 37°C and 200 rpm and shake sample for 1 h to recover cells.
  g. Spin cells down at 3,000 g for 3 min at 20°C–22°C.
  h. Discard supernatant and resuspend cells in 50 μL of LB media.
  i. Plate cells on LB agar plates that contain 50 μg/mL kanamycin.
  j. Place LB/Kanamycin plates in a 37°C incubator for a minimum of 16 h. Colonies should be visible after 24 h.
7. Express His-DYRK1a.
  a. Add 50 μL of 50 mg/mL kanamycin to Erlenmeyer flask that contains 50 mL of LB media, so that the final concentration of kanamycin in the LB media is 50 μg/mL.
  b. Inoculate the 50 mL LB/Kanamycin flask with cells from a colony on the plate from step 6j.
  c. Grow starter culture in a shaking incubator 16–18 h at 37°C with shaking at 200 rpm.
  d. Inoculate two 1 L terrific broth (TB) cultures with 10 mL starter culture and 50 μg/mL kanamycin.
  e. Grow 1 L cultures at 37°C with shaking at 200 rpm until the OD reaches 1.0.
  f. Induce cultures with IPTG so that the final concentration of IPTG in each 1 L culture is 0.5 mM and reduce the temperature of the shaker to 16°C to promote proper protein folding during expression.
  g. Let cultures incubate in the shaker 16–18 h at 16°C and 200 rpm.
  h. Pellet cells at by centrifugation 5,500 g for 25 min at 4°C.

***Note:*** Store pellet at −20°C 16–18 h or flash freeze it for long-term storage at −80°C if immediate kinase purification is not required.

**Figure 2:**
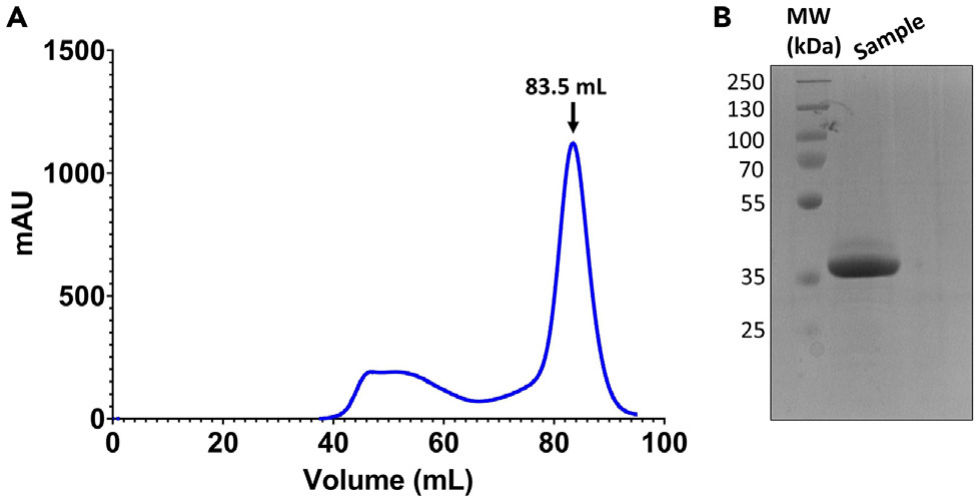
DYRK1a kinase purification (A) Size-exclusion chromatogram for DYRK1a kinase run on a Superdex 200 column (GE). (B) SDS-PAGE of pooled DYRK1a kinase after running size-exclusion chromatography. Sample was run on a 15% gel.

8. Lyse cells.
  a. Resuspend cells from both pellets in 50 mL DYRK1a lysis buffer.
    ***Note:*** When using TB media, the cell pellets are typically larger than the cell pellets produced from LB media. Thus, you can use more lysis buffer to resuspend the cell pellet if needed. Since the His-DYRK1a will be initially purified by Ni-NTA affinity resin, the final volume of the cell lysate does not affect this purification step.
  b. Sonicate resuspended cells with five 30 s cycles. For each 30 s cycle, set the on time to 1 s, the off time to 5 s, and the amplitude to 90 A. Let the sample rest 3 min on ice between cycles.
    ***Note:*** Keep the sample in ice throughout the sonication process to prevent the sample from heating up during sonication.
  c. Pellet cell lysate by centrifugation at 15,000 g for 35 min at 4°C.
9. Purify His-DYRK1a with nickel affinity resin.
  a. Equilibrate 5 mL of Ni-NTA beads with 20 CV DYRK1a lysis buffer.
    ***Note:*** Ni-NTA column purification must be done at 4°C.
  b. Run supernatant from step 8c over the pre-equilibrated nickel beads.
  c. Collect the flowthrough and pass it through the nickel beads a second time.
  d. Wash the column with 10 CV of DYRK1a wash buffer.
  e. Elute protein with 4 CV of DYRK1a elution buffer.
  f. Run SDS-PAGE gel to confirm purity as shown in Figure 2B.
10. Further purify His-DYRK1a CTD by size-exclusion chromatography.
  a. Concentrate eluted protein to 5 mL using a protein concentrator with a molecular weight cut off of 10 kDa.
  b. Buffer exchange eluted protein to DYRK1a SEC buffer by adding 5 mL of SEC buffer to concentrator at a time and reconcentrating the sample to 5 mL.
  c. Repeat step 10 b at least 4 additional times to ensure that a minimum of 25 mL of CTD SEC buffer is used to minimize the concentration of imidazole in solution before loading onto the column.
  d. Concentrate the buffer exchanged sample to 2 mL.
  e. Equilibrate Superdex 200 size-exclusion column (GE) with DYRK1a SEC buffer.
  f. Run sample over SEC and pool fractions from peak corresponding to ∼40 kDa. An example chromatogram is shown in Figure 2A.
  g. Check protein concentration and concentrate as needed.

***Note:*** For this protocol, you do not need a super concentrated kinase sample. A concentration of 0.5 mg/mL is sufficient.

### Purify ERK2 kinase

**Timing: 5 days**

This step is an example protocol for purifying an active CTD kinase that will be used to phosphorylate the CTD substrate for the pull-down experiment.

11. Transform the ERK2/MEK1 pET-28a plasmid into *E. coli* BL21 (DE3) cells.
  a. Add 50 ng of the ERK2/MEK1 plasmid to a 50 μL aliquot of *E. coli* BL21 (DE3) cells.
  b. Incubate plasmid-cell solution on ice for 30 min.
  c. Place tube containing the plasmid-cell solution in a water bath set to 42°C for 60 s to heat shock.
  d. Place tube on ice for 5 min after heat shock.
  e. Transfer heat shocked cells to a microcentrifuge tube containing 800 μL SOC media.
  f. Spin cells down at 3,000 g for 3 min at 20°C–22°C.
  g. Discard supernatant and resuspend cells in 50 μL of LB media.
  h. Plate cells on LB agar plates that contain 100 μg/mL ampicillin.
  i. Place LB/Ampicillin plates in a 37°C incubator for a minimum of 16 h. Colonies should be visible after 24 h.
12. Express ERK2/MEK1.
  a. Add 100 μL of 50 mg/mL ampicillin to Erlenmeyer flask that contains 50 mL of LB media, so that the final concentration of ampicillin in the LB media is 100 μg/mL
  b. Inoculate the 50 mL LB/Ampicillin flask with cells from a colony on the plate from step 11j.
  c. Grow starter culture in a shaking incubator 16–18 h at 37°C with shaking at 200 rpm.
  d. Inoculate two 1 L Luria-Bertani broth (LB) cultures with 10 mL starter culture and 50 μg/mL ampicillin.
  e. Grow 1 L culture at 37°C with shaking at 200 rpm until the OD reaches 0.6.
  f. Induce cultures with IPTG so that the final concentration of IPTG in each 1 L flask is 0.5 mM.
  g. After induction cultures were incubated at 37°C for 4 h with shaking at 200 rpm.
  h. Pellet cells by centrifugation at 5,500 g for 25 min at 4°C.

***Note:*** Store pellet at −20°C for 16–18 h or flash freeze it for long-term storage at −80°C if immediate kinase purification is not required.

**Figure 3:**
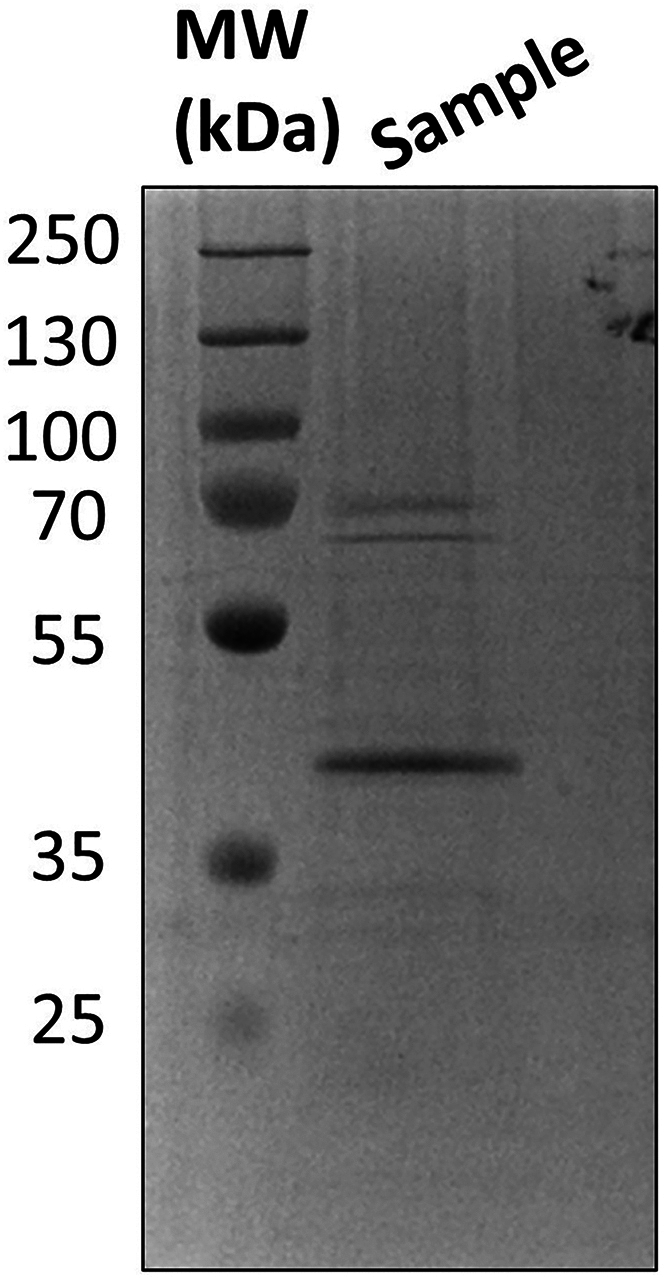
SDS-PAGE of pooled ERK2 kinase after eluting the to sample from a Mono Q column Sample was run on a 15% gel.

13. Lyse cells.
  a. Resuspend cell pellet in 50 mL ERK2 lysis buffer.
  b. Sonicate resuspended cells with five 30 s cycles. For each 30 s cycle, set the on time to 1 s, the off time to 5 s, and the amplitude to 90 A. Let the sample rest 3 min on ice between cycles.
    ***Note:*** Keep the sample in ice throughout the sonication process to prevent the sample from heating up during sonication.
  c. Pellet cell lysate by centrifugation at 15,000 g for 35 min at 4°C.
14. Purify ERK2 with nickel affinity resin.
  a. Equilibrate 5 mL of Ni-NTA beads with 20 CV ERK2 lysis buffer.
    ***Note:*** Ni-NTA column purification can be done at 20°C–22°C.
  b. Run supernatant from step 13c over the equilibrated nickel beads.
  c. Collect the flowthrough and run the flowthrough over the nickel beads a second time.
  d. Wash the column with 10 CV of ERK2 lysis buffer.
  e. Elute protein with 4 CV of ERK2 elution buffer.
  f. Run SDS-PAGE gel to confirm purity.
15. Further purify ERK2 by anion exchange chromatography.
  a. Prepare a dialysis bag from snakeskin dialysis tubing (10,000 MWCO) and transfer the eluted protein into the membrane bag.
  b. Put dialysis bag in 1 L of ERK2 Mono Q buffer A and dialyze for 16–18 h at 4°C.
  c. Equilibrate Mono Q column (GE Life Sciences) with ERK2 Mono Q buffer A.
    ***Note:*** ERK2 was co-expressed with MEK1 (R4F). ERK2 has an N-terminal hexahistidine tag, while MEK1 (R4F) does not, thus most of the MEK1 (R4F) should be separated from the desired ERK2 protein during the previous Ni-NTA affinity purification. However, as an additional polishing step we do anion exchange. Both ERK2 and MEK1 (R4F) are approximately 41.0 kDa, thus size-exclusion chromatography will not be useful in this instance. However, the predicted isoelectric point of ERK2 (pI = 6.50) is slightly less acidic than that of MEK1 (pI = 6.03), thus anion exchange chromatography can be used in this case to remove any trace amounts of MEK1 (R4F) that remain in the sample.
  d. Load dialyzed sample on Mono Q column and elute protein with a 50–1000 mM NaCl gradient using ERK2 Mono Q buffer B.
  e. Run SDS-PAGE gel to confirm purity as shown in Figure 3. A band for ERK2 should appear at ∼41 kDa.
  f. Pool fractions containing ERK2. Check protein concentration and concentrate as needed.
    ***Note:*** For this protocol, you do not need a super concentrated kinase sample. Concentrating to 0.5 mg/mL is sufficient.

**Figure 4:**
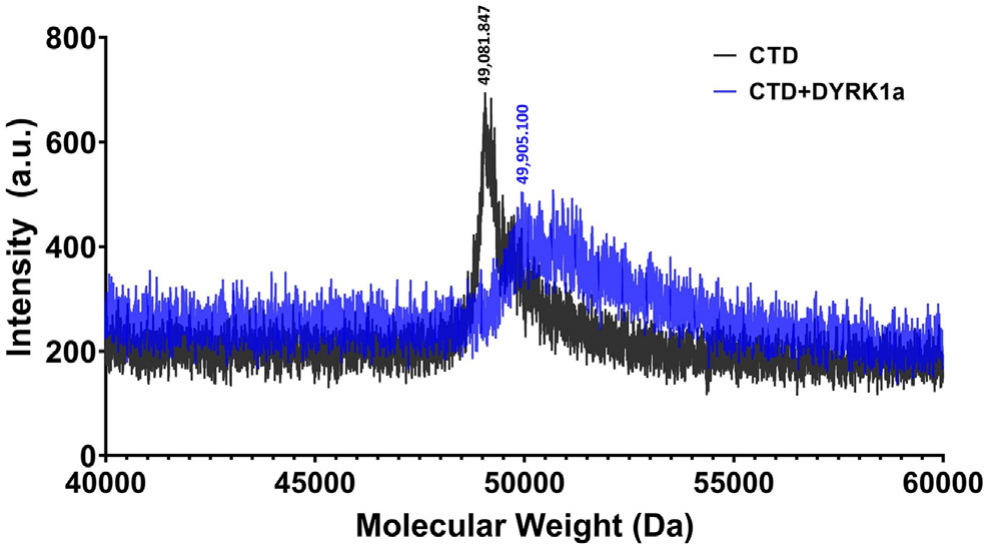
MALDI-TOF spectra for an untreated CTD sample (black) and a DYRK1a-treated CTD (blue) The molecular weights for the highest peak is shown for each spectra. The difference between the mass of the kinase treated sample versus the untreated sample suggests that approximately 10 phosphate groups were added to the CTD in the treated sample.

### Prepare CTD substrate

**Timing: 1 day**

This step details conditions to site-specifically phosphorylate the CTD substrate *in vitro* in preparation for a pull-down experiment.

16. Treat CTD with kinase.
  a. Set up 120 μL kinase reactions as shown in the table below. Be sure to set up a control sample in parallel with the treated sample where 30 μL of ddH_2_O is added to the reaction mix in place of the kinase.
    ***Note:*** 0.4 mg/mL kinase was added to the kinase reaction (for final concentration of 0.1 mg/mL, regardless of the kinase used).

**Table:** Kinase reaction mix

Reagent	Amount
4× kinase buffer	30 μL
Kinase (0.4 mg/mL)	30 μL
HGST-26X CTD (4 mg/mL)	30 μL
ATP (8 mM)	30 μL

    ***Note:*** The final reaction volume should be 120 μL, where 100 μL will be used in the pull-down experiment and 20 μL will be saved to confirm phosphorylation by mass spectrometry and gel electrophoresis. Example MALDI-TOF mass spectra of untreated and kinase-treated samples are shown in Figure 4.
  b. Pipette up and down thoroughly to ensure the reaction solution is well mixed.
  c. Place reaction tubes into an Eppendorf thermomixer C. Set shaking to 600 rpm and the temperature to 30°C.
  d. The reaction time varies based on kinase activity. See table below for suggested duration of kinase reaction.

**Table:** Kinase reaction mix

Kinase	Specificity	Time
DYRK1a	pS2	16–18 h
CDK7	pS5	16–18 h
ERK2	pS5	3 h
17. Prepare GST beads.
  a. Pipette 200 μL of a slurry of 50% glutathione beads into a microcentrifuge tube.
    ***Note:*** You want a 1:1 ratio of GST bead volume to kinase reaction solution, thus if you plan to add 100 μL kinase reaction mix to the beads then you will want 200 μL of 50% beads.
    i. You will need 1 tube of beads per sample. Use a fresh pipette tip to pipette beads into each tube to ensure you are as accurate as possible when pipetting the beads, since they are very sticky!
  b. Centrifuge beads at 4,000 g for 2 min at 20°C–22°C and remove the supernatant.
    **CRITICAL:** Be careful not to disturb the beads.
  c. Wash the beads by adding 1 mL of buffer C to each tube and incubating the tubes on a nutator for 5 min at 4°C.
    i. Wash the beads a total of 3 times. Spin the beads down as done in step 17b after each wash.
  d. After all washes are complete carefully remove the supernatant from the beads.
  e. Add 100 μL of the phosphorylated HGST-26X CTD mix to the washed beads. The remaining 20 μL of the HGST-26X CTD can be stored at −20°C until mass spectrometry can be done to confirm phosphorylation.
  f. Increase the final volume of the solution in each tube to 1 mL by adding approximately 800 μL of buffer C.
  g. Bind HGST-26X CTD to the GST beads by incubating the tubes on a nutator 16–18 h at 4°C.
    ***Note:*** Use snap-cap tubes for this to ensure that your samples do not open overnight.

### Prepare nuclear lysate

**Timing: 1 day**

This section details steps to extract nuclear lysate from HEK293 cells to be used as the “prey” in a pull-down experiment. Since RNA polymerase II is a nuclear protein complex, we used nuclear lysate for our pull-down experiment to reduce the appearance of hits that are not biologically relevant.

18. Pellet cells prepared in the before you begin section by spinning at 200 g for 5 min. Discard PBS supernatant.
19. Extract cytoplasmic proteins.
  a. Resuspend washed cells in 1 PCV (approximately 1 mL) of buffer A.
  b. Vortex tubes and incubate on ice for 15 min.
  c. Centrifuge tubes at 15,000 g for 10 min at 4°C.
  d. Carefully remove the supernatant from each tube and save it as the cytoplasmic fraction.
20. Extract nuclear proteins.
  a. Gently resuspend cell debris in 1 PCV (approximately 1 mL) of buffer A by pipetting up and down a few times.
  b. Spin down at 15,000 g for 1 min at 4°C.
  c. Discard the supernatant.
  d. Resuspend cell debris in 1 PCV of buffer B and supplement solution with a 1:1000 dilution of benzonase to reduce protein:DNA interactions.
    i. Example: If the approximate volume of the solution is 1,200 μL add 1.2 μL of benzonase.
  e. Incubate the lysate/benzonase solution at 20°C–22°C for 1 h.
  f. Centrifuge tubes at 15,000 g for 10 min at 20°C–22°C.
  g. Collect the supernatant as the nuclear fraction.

### Pull-down

**Timing: 1–2 days**

This step details how to set up the pull-down experiment with HEK293 nuclear lysate “prey” and the phosphorylated CTD “bait.”

21. Wash the GST beads after incubating with HGST-26X CTD.
  a. Wash the GST beads by adding 1 mL of buffer C to each tube and incubating the tubes on a nutator for 5 min at 4°C. Wash the CTD-bound GST beads a total of 3 times. Spin the beads down as done in step 17b after each wash.
  b. After all washes are complete carefully remove the supernatant from the beads.
22. Set up pull-down.
  a. Split nuclear protein extract evenly between samples.
    i. For example, if you have two samples, divide the nuclear protein extract equally by adding 500 μL extract to each tube with beads. Since 200 million cells were lysed, each sample should contain lysate from approximately 100 million cells. For optimal results, aim for 50 million cells per sample. In a standard CTD pull-down experiment, split the sample evenly among four tubes, comprising two control samples and two treated samples.
  b. After adding nuclear protein lysate to CTD-bound beads, incubate the tubes on a nutator for 24–48 h at 4°C.

***Note:*** We typically incubate for 48 h.

23. Wash pull-down samples and elute in preparation for MS/MS.
  a. Centrifuge samples at 4,000 g for 2 min at 4°C and carefully remove the supernatant.
  b. Wash the beads by adding 1 mL of low salt buffer to each tube and incubate the tubes on a nutator for 5 min at 4°C.
    i. Wash the CTD-bound beads a total of 2 times with the low salt buffer. Spin the beads down as done in step 17b after each wash.
  c. Wash the beads by adding 1 mL of high salt buffer to each tube and incubate the tubes on a nutator for 5 min at 4°C.
    ***Note:*** We used two buffers with different ionic strengths to help reduce nonspecific binding interactions.
    i. Wash the CTD-bound beads a total of 3 times with the high salt buffer. Spin the beads down as done in step 17b after each wash.
  d. Add 100 μL of elution buffer and incubate the tubes on a nutator for at least 2 h at 4°C.
  e. Centrifuge tubes at 4,000 g for 2 min at 4°C.
  f. Collect the supernatant as your pull-down samples for MS/MS analysis.
    ***Note:*** Nuclear lysates can be stored at −20°C for up to a week.

## Expected outcomes

After submitting samples for MS/MS analysis, you will obtain a table of proteins hits with corresponding peptide-spectrum match (PSM) counts for each hit in every sample as shown in Table 1.

**Table 1:** Example of output count files from the pull-down experiment

Protein	Control 1	Control 2	Kinase-treated 1	Kinase-treated 2
Q9UKV3	1	1	4	5
O96019	–	–	3	6
O43823	8	7	24	23
Q9ULX6	13	10	112	100
Q66PJ3	2	4	–	–
Q96GD4	–	–	5	6
O75531	8	4	60	53
Q9NRL2	3	3	9	11
Q9UIG0	18	14	15	16
Q9UIF9	2	4	60	65

**Figure 5:**
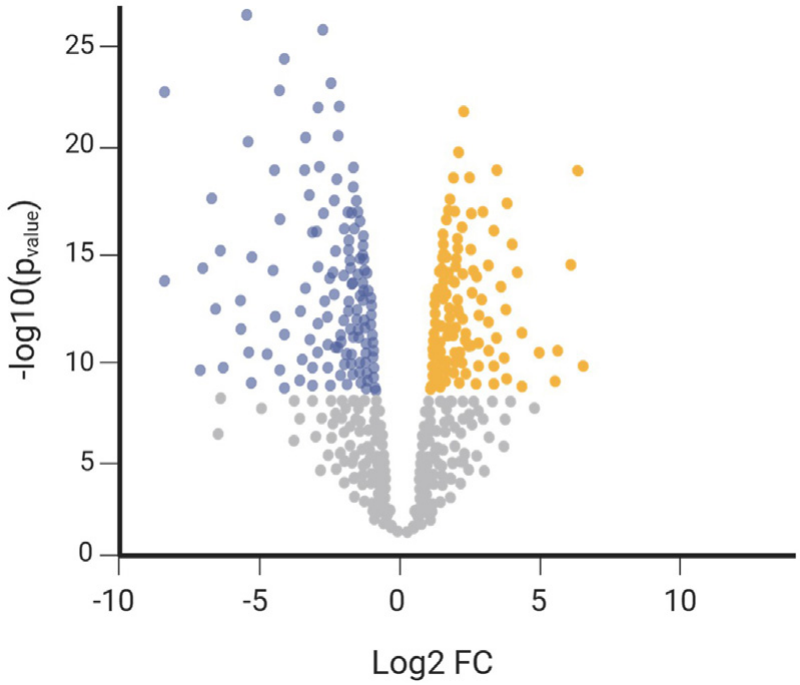
Example volcano plot that compares hits from control versus kinase-treated sample Figure created with BioRender.com.

From these data, the log2 fold change (FC) and −log10 *p* value can be calculated and plotted in a volcano plot as shown in Figure 5.

## Quantification and statistical analysis

Eluted proteins from step 23f were prepared for tandem MS as stated in Moreno et al.^1^ Briefly, proteins were separated using a 75 μM × 25 cm Acclaim PepMap100 C-18 column (Thermo Scientific) and analyzed online by nanoelectrospray-ionization tandem MS on a Thermo Scientific Fusion Tribrid Orbitrap mass spectrometer. MS1 scans were collected at high resolution, and MS2 scans were acquired in the ion trap in rapid scan mode with fragmenting by collision-induced dissociation. Proteome Discoverer 2.3 (Thermo Scientific) was used to identify proteins by searching against the human reference proteome for UniProt. A false discovery rate of 1% was used to identify peptides, and a z-score was calculated to estimate the changes in protein abundance between control and kinase-treated samples according to Floyd et al.^23^

Counts from the two control replicates and the two kinase-treated replicates were imputed to generate missing values. Then the data were quantile normalized and log2 transformed. *p* values were calculated using a two-tailed t-test, and significantly enriched proteins were defined as having a *p* value of 0.05 or lower. A volcano plot was generated by plotting the log2 fold change versus the -log10 of the calculated *p* value in GraphPad Prism (version 10.2.3). See supplementary table 1 in Moreno et al. for an example of expected results.^1^

## Limitations

The use of kinases to phosphorylate the CTD of RNAPII *in vitro* relies on previous well-characterized kinases for their specificity and kinase activity. Of the known CTD kinases, we currently have the ability to phosphorylate Ser2 (by DYRK1a), Ser5 (by CDK7, CDK9, or ERK2), or Tyr1 (by c-Abl).^20^^,^^21^^,^^22^^,^^24^^,^^25^ Thus, our ability to discern hits for different CTD post-translational modifications is limited to the phosphorylation patterns that we can recapitulate *in vitro.* Other strategies must be employed to characterize the interactomes of CTD PTMs, such as pThr4, until a kinase is identified that can place these CTD PTMs *in vitro*. Additionally, our strategy used a 26X WT CTD mostly of consensus sequence as the substrate for our pull-down experiment. However, the human 52X CTD might recruit additional factors since some heptads deviate from the consensus sequence. Interactomes for divergent sequences have yet to be studied but can be easily attained with our strategy. Finally, our experimental design leverages the proteomics experiment described in this protocol to identify lead RNAPII-binding proteins. The output of our approach yields a list of hundreds of lead proteins that must further be characterized via other methods, such as those shown in Moreno et al., to validate the hit as a RNAPII-binding protein.^1^

## Troubleshooting

### Problem 1

Contaminating skin, cytosolic, or mitochondrial proteins appear as hits in final result table.

### Potential solution

No matter how careful you are when doing the nuclear extraction steps, contaminating proteins can appear in the raw output data. To mitigate this issue, we suggest using the ID mapping function on the UniProt website to quickly identify the subcellular location of each protein, so that you can filter out cytosolic, mitochondrial, or secreted proteins from your list of hits.

### Problem 2

There is a little difference between the wild-type and phosphorylated PSM counts for hit proteins.

### Potential solution

Ensure that kinase used to treat the CTD substrate is active. You can run an electrophoretic mobility shift assay (EMSA) to check for kinase activity or use matrix-assisted laser desorption ionization- time of flight (MALDI-TOF) mass spectrometry to determine the approximate number of phosphorylation groups that were added to the 26X CTD substrate. If fewer than 10 phosphate groups are added to the CTD substrate, this might not be enough to see differential protein recruitment in a pulldown experiment.

## Resource availability

### Lead contact

Further information and requests for resources and reagents should be directed to and will be fulfilled by the lead contact, Dr. Yan Jessie Zhang (jzhang@cm.utexas.edu).

### Technical contact

Technical questions on executing this protocol should be directed to and will be answered by the technical contact, Haley A. Hardtke (hhardtke@utexas.edu).

### Materials availability

There are no unique reagents associated with this protocol.

### Data and code availability

This protocol does not report any original code.

## Acknowledgments

This work was supported by grants from the 10.13039/100000002National Institutes of Health (R01GM104896 R01GM125882 to Y.J.Z. and R35 GM148356 to Y.J.Z.) and L. Leon Campbell Professorship Funds.

Proteomics data were acquired in the UT Austin Center for Biomedical Research Support Biological Mass Spectrometry Facility (RRID:SCR_021728). The graphical abstract was created with BioRender.com.

## Author contributions

Methodology, R.Y.M. and H.A.H.; writing – original draft, H.A.H.; writing – review and editing, R.Y.M. and Y.J.Z.; supervision and funding acquisition, Y.J.Z.

## Declaration of interests

The authors declare no competing interests.
